# Non-Gaussian Analysis of Diffusion Weighted Imaging in Head and Neck at 3T: A Pilot Study in Patients with Nasopharyngeal Carcinoma

**DOI:** 10.1371/journal.pone.0087024

**Published:** 2014-01-23

**Authors:** Jing Yuan, David Ka Wai Yeung, Greta S. P. Mok, Kunwar S. Bhatia, Yi-Xiang J. Wang, Anil T. Ahuja, Ann D. King

**Affiliations:** 1 Department of Imaging and Interventional Radiology, The Chinese University of Hong Kong, Shatin, New Territories, Hong Kong, China; 2 Shenzhen Research Institute, The Chinese University of Hong Kong, Shenzhen, Guangdong, China; 3 Department of Electrical and Computer Engineering, University of Macau, Taipa, Macau SAR, China; University of California San Francisco, United States of America

## Abstract

**Purpose:**

To technically investigate the non-Gaussian diffusion of head and neck diffusion weighted imaging (DWI) at 3 Tesla and compare advanced non-Gaussian diffusion models, including diffusion kurtosis imaging (DKI), stretched-exponential model (SEM), intravoxel incoherent motion (IVIM) and statistical model in the patients with nasopharyngeal carcinoma (NPC).

**Materials and Methods:**

After ethics approval was granted, 16 patients with NPC were examined using DWI performed at 3T employing an extended b-value range from 0 to 1500 s/mm^2^. DWI signals were fitted to the mono-exponential and non-Gaussian diffusion models on primary tumor, metastatic node, spinal cord and muscle. Non-Gaussian parameter maps were generated and compared to apparent diffusion coefficient (ADC) maps in NPC.

**Results:**

Diffusion in NPC exhibited non-Gaussian behavior at the extended b-value range. Non-Gaussian models achieved significantly better fitting of DWI signal than the mono-exponential model. Non-Gaussian diffusion coefficients were substantially different from mono-exponential ADC both in magnitude and histogram distribution.

**Conclusion:**

Non-Gaussian diffusivity in head and neck tissues and NPC lesions could be assessed by using non-Gaussian diffusion models. Non-Gaussian DWI analysis may reveal additional tissue properties beyond ADC and holds potentials to be used as a complementary tool for NPC characterization.

## Introduction

Diffusion-weighted imaging (DWI) is capable of noninvasively measuring water diffusivity in living tissues [1]. In the traditional diffusion theory, the displacement of freely mobile water molecules diffusing from one location to another in a certain time is considered to have a Gaussian distribution. Based on this Gaussian diffusion behavior, a mono-exponential decay function of DWI signal intensity with regard to the increase of b-value has been adopted for diffusion analysis in most clinical studies [2]. Apparent diffusion coefficient (ADC) can be quantified to evaluate the average diffusivity of tissues. Because microstructural changes at the cellular level in tissues due to pathology may hinder the motion of water molecules and consequently affect ADC, DWI is growing rapidly in scope and importance for various clinical applications.

However, water diffusion behavior in biological tissues is much more complicated than the completely free water diffusion in space due to the complex cellular structures of tissues with many diffusion barriers like membranes. Consequently, the displacement probability of tissue water may substantially deviate from a Gaussian form and hence violates the validity of the mono-exponential model to some extent. Therefore, several advanced diffusion models (referred to as non-Gaussian diffusion models here) have been proposed to account for the non-Gaussian diffusion behavior of biological tissues to allow a more comprehensive analysis of DWI data [3]–[8].

Introvoxel incoherent motion (IVIM) was proposed by Le Bihan et al [7], [8] to account for the effect of capillary perfusion on the aggregate DWI signal, In this IVIM model, two separate parameters (along with their volume fractions) have been proposed to reflect the true tissue diffusivity and capillary perfusion, respectively, in the mathematical form of a bi-exponential decay function. By considering the continuous distribution of microscopic diffusion compartments attenuating at different rates with b-values, stretched-exponential model (SEM) [6] and statistical diffusion model (SDM) were also proposed [5]. Besides, diffusion kurtosis imaging (DKI) model used the Taylor expansion of the DWI signal attenuation in powers of b-value to measure the excess diffusion kurtosis in addition to the diffusion coefficient [4].

Non-Gaussian diffusion models were firstly proposed and primarily applied for brain DWI [3]–[7], [9]–[20] while their applications to other tissues, including head and neck, have been increasing in recently years [21]–[24]. In fact, the normal mono-exponential DWI model is still dominant in clinical head and neck applications such as tumor detection, staging, characterization as well as treatment response prediction [25]–[35]. In principle, non-Gaussian diffusion models may better describe the complicated water diffusion behavior in living tissues and may provide additional information on tissue heterogeneity, vascularity and cellularity beyond ADC. However, the previous non-Gaussian head and neck diffusion studies [21]–[24] only focused on a specific non-Gaussian diffusion model. As such, studies on comprehensive DWI analysis using different non-Gaussian models in the head and neck are still sparse. More importantly, different non-Gaussian diffusion models may reveal different aspects of tissue properties. Thus their roles in different tissue and lesion characterization should be individually evaluated and compared so as to find the most suitable method for a specific application. In this pilot study, we aimed to explore the technical feasibility and compare results of different non-Gaussian diffusion models (DKI, IVIM, SEM and SDM), for head and neck DWI analysis in the patients with nasopharyngeal carcinoma (NPC). We investigated whether non-Gaussian diffusion behavior could be identified and characterized by non-Gaussian DWI analysis. We also intended to technically discover which non-Gaussian diffusion model best fit the DWI signal decay in normal tissues and NPC lesions by evaluating the goodness-of-fit, and to compare non-Gaussian DWI model with the normal mono-exponential model.

## Methods

### Patients

Institutional ethical review board of the Chinese University of Hong Kong approved this study and written informed consents were provided from each participant. Sixteen patients (four females and twelve males; mean age of 57.5 years) with NPC who had newly diagnosed head and neck cancer were recruited. The primary tumors were confirmed by biopsy, while lymph node metastases were diagnosed on imaging criteria.

### MRI Examination

All MRI scans were performed on a 3T MRI scanner (Achieva, Philips Medical Systems, Best, The Netherlands) using a receive-only 16-channel head and neck array coil. DWI examination was performed by using a fat-suppressed single shot spin echo EPI (SS-SE-EPI) sequence with a pair of rectangular diffusion gradient pulses along all three orthogonal axes to obtain isotropic DWI images. Imaging parameters were: TR/TE  =  1561/46 ms, flip angle (FA)  =  90°, number of signal average (NSA)  =  3, field of view (FOV)  =  230 mm, matrix size  =  136×109, slices  =  5, slice thickness  =  4 mm, Sensitivity encoding (SENSE) factor  =  2, 12 b-factors: 0, 100, 200, 300, 400, 500, 600, 800, 1000, 1200, 1400 and 1500 s/mm^2^. These b-factors were selected by taking account of the acceptable SNR, reasonable total scan time and specific requirements for DWI analysis using different Non-Gaussian models. The low b-factors below 200 s/mm^2^ were of importance to capture the much higher pseudo-diffusion due to capillary perfusion for IVIM analysis. The high b-factors over 1000 s/mm^2^ were necessary for DKI analysis for K_DKI_ quantification because the DKI model based non-Gaussianality of diffusion could only be revealed at high b-factors [4]. Much more medium valued b-factors (between 200 and 1000 s/mm^2^) were applied for the accurate quantification of ADC and D_IVIM_, otherwise their estimates may be biased by the inclusion of low and high b-factors during fitting. In order to enhance the SNR and thus quantification accuracy, in particular for high b-factors, a NSA of three was utilized but at the cost of longer scan time. The total DWI scan time was about six minutes. As tissues and lesions in head and neck do not necessarily reflect the structures with apparent orientation, diffusion in head and neck may not show anisotropic properties so diffusion-tensor imaging (DTI) was not conducted. T_2_-weighted anatomical images were also acquired using a fat-suppressed turbo spin echo (TSE) sequence (TE/TR  =  80/4500 ms, echo train length  =  15, FOV  =  230 m, matrix  =  364×262, NSA  =  2, SENSE factor  =  2).

### DWI Analysis

DWI images were first registered to the baseline image with b = 0 s/mm^2^ on the MRI console to compensate for possible patient motion, then exported and processed off-line. All non-Gaussian diffusion quantification and analysis were conducted by using an in-house developed Matlab program (version 7.9, The MathWorks, Inc, Natick, MA, USA). The traditional mono-exponential model and four non-Gaussian diffusion models were mathematically expressed as follows:

1. Traditional mono-exponential diffusion model
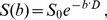
(1)


where S(b) and S_0_ denote the signal intensity obtained with the diffusion gradient b-value of b and zero, respectively, for all DWI models. *D* is the apparent diffusion coefficient (ADC), the sole parameter to be estimated through fitting to this model.

2. Diffusion kurtosis imaging (DKI) model [4]

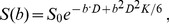
(2)


where D is the diffusion coefficient and K denotes diffusional kurtosis, a dimensionless parameter reflecting the deviation of diffusion distribution from the Gaussian form.

3. Intravoxel incoherent motion (IVIM) model [7], [8]

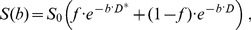
(3)


where *f* is the unit-less perfusion volume fraction. D* is the pseudo-diffusion coefficient influenced by the tissue microcapillary perfusion, and *D* is the true diffusion coefficient.

4. Stretched exponential model (SEM) [6]

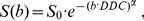
(4)


where *DDC* is the distributed diffusion coefficient and α is the stretching parameter or heterogeneity index without unit (0≤ α≤1).

5. Statistical diffusion model (SDM) [5]

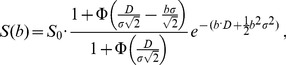
(5)


where *D* is the center and σ is the width of the Gaussian distribution of the diffusion coefficient. *Φ* denotes the error function which is defined as:
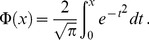
(6)


As each model above includes its own diffusion coefficient, these diffusion coefficients are denoted as D with the subscript of the abbreviated model name like *D_IVIM_* and *D_DKI_*, to avoid ambiguity hereafter. Note that D_mono_ is equivalent to ADC.

Region-of-interests (ROIs) were drawn manually on both DWI images with b  =  0 s/mm^2^ and T2-weighted TSE anatomical images through the slice with the maximum area of primary tumors and metastatic nodes by an MRI physicist (JY) and then checked and confirmed by a radiologist (ADK) with over 15 years’ experience in head and neck radiology. Diffusion attenuated signal intensities within ROIs were fitted against b-values according to the mono-exponential and non-Gaussian diffusion models as shown in Eq. [1]–[5] using the non-linear least-square fitting based on Levenberg-Marquardt algorithm. Maps of diffusion coefficients and other non-Gaussian parameters within the ROIs were generated. In particular for IVIM model, DWI data fitting was performed using the asymptotic fitting method, which provided more accurate and robust estimation than the full fitting of DWI signals to the bi-exponential function [36]. In detail, D_IVIM_ was first obtained with a least-square fitting to a mono-exponential function by using the data points at b-values over 200 s/mm^2^. The fitted curve was then extrapolated to obtain an intercept at b = 0. The ratio between this intercept and the DWI data point at b  =  0, gave an estimate of *f_IVIM_*. Finally, the obtained D_IVIM_ and f_IVIM_ were substituted into Eq. 3 and non-linear least-square fitted against all b-factors to estimate D*. To evaluate the goodness of fit by each model, adjusted coefficient of determinant (R^2^) that accounts for the number of degree of freedom (DOF) were calculated and compared. The poorly fitted parameter value for the voxels with R^2^<0.8 was set as zero. To test if non-Gaussian diffusion models provided significantly better fits than the mono-exponential model, an F test [37] was performed with a significance level p-value of 0.05. The correlations between the voxel-wise non-Gaussian diffusion coefficients and the mono-exponential ADC within the lesion ROIs were evaluated using Spearman rank-order coefficient. Mean value and standard deviation of ADC and non-Gaussian parameters were calculated for primary tumors and metastatic nodes. Mann-Whitney *U*-test was performed to determine if there was any statistically significant difference between DWI parameters extracted from primary tumors and those from metastatic nodes using a cut-off p-value level of 0.05.

## Results

DWI examination was successful in all patients without serious motion artifact that might hinder subsequent data analysis. Thirteen primary tumors were identified. The sizes of these thirteen primary tumors measured from T2-weighted TSE anatomical images ranged from 120.5 mm^2^ to 1228.6 mm^2^ with a mean value of 571.1 mm^2^. In three patients, the primary tumors have been excluded for the analysis due to their small sizes (<5 mm in the minimum diameter). Nine metastatic nodes were also identified and included in the analysis. The sizes of these metastatic nodes measured from T2-weighted TSE anatomical images ranged from 87.3 mm^2^ to 886.8 mm^2^ with a mean value of 425.9 mm^2^.

**Figure 1:**
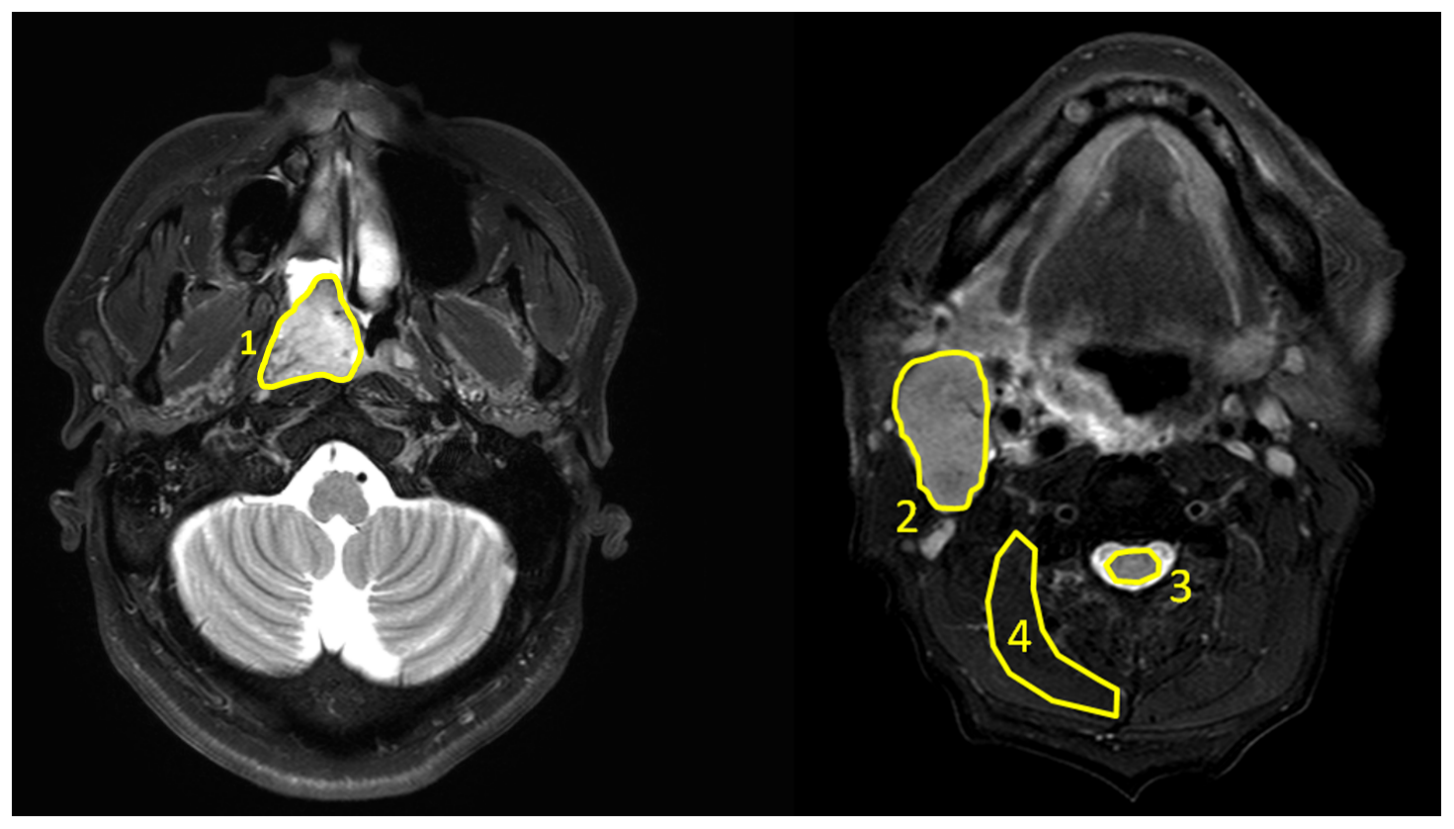
T2-weighted fat-suppressed fast-spin-echo anatomical images. Regions of interest (ROIs) were drawn on primary tumors of nasopharyngeal carcinoma (NPC), metastatic node, spinal cord and muscle and labeled as one to four respectively.

**Figure 2:**
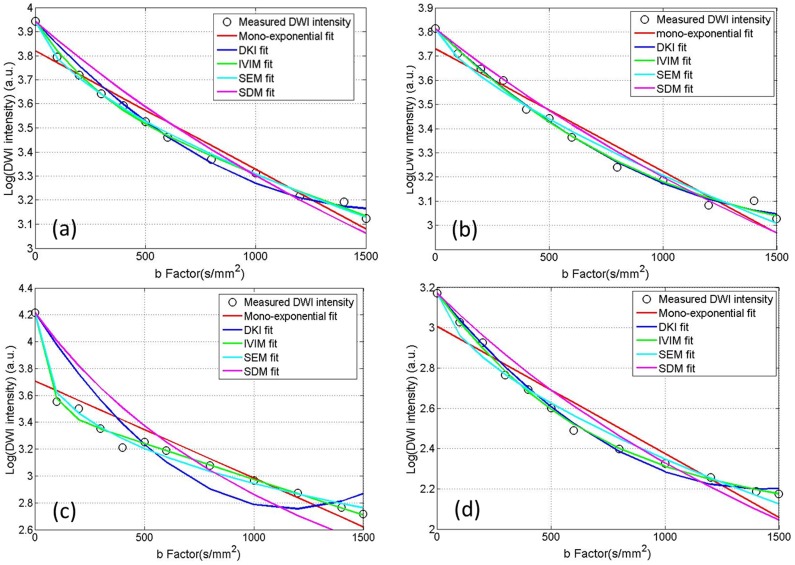
The measured DWI signals and the best-fit curves within the primary tumor (a), metastatic node (b), spinal cord (c) and muscle (d) according to the mono-exponential and non-Gaussian diffusion models. The measured DWI signals showed substantial non-Gaussian diffusion behaviors and could be significantly better fitted by non-Gaussian models.

Figure 1 shows the T_2_-weighted TSE anatomical images of a NPC primary tumor (a) in one patient and a metastatic node (b) in another patient. The ROIs labeled as 1 to 4 in Fig. 1 depict primary tumor, metastatic node, spinal cord and muscle for DWI signal decay curve analysis, respectively. The averaged DWI signals within the ROIs 1–4 and the best fit curves to different diffusion models were shown in Fig. 2a-d. As seen in Fig. 2, diffusion attenuated signals in all four tissues showed substantial deviation from the mono-exponential attenuation, indicating non-Gaussian diffusion behavior when using an extended b-value range up to 1500 s/mm^2^. Compared to the mono-exponential model, IVIM and SDM model showed significantly better fitting (p<0.05) for all four tissues. DKI model yielded significantly better fitting (p<0.05) in primary tumors, nodes, muscles but not in spinal cords compared to the mono-exponential model. On the other hand, SDM model just obtained comparable goodness of fit (slightly higher R^2^ in primary tumors, metastatic nodes, muscles but slightly lower R^2^ in muscles) but not significantly better fitting than the mono-exponential model. In our patient cohort, the fitting results showed that all primary tumors and metastatic nodes exhibited non-Gaussian diffusion behaviors when an extended b-value range was employed, indicating that significantly better fitting of DWI data could be achieved using non-Gaussian DWI models.

**Figure 3:**
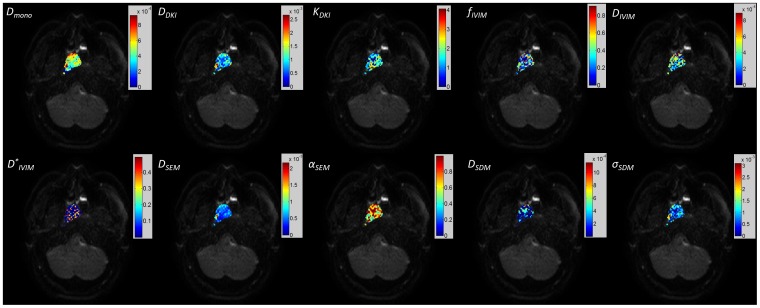
Maps of the mono-exponential apparent diffusion coefficient (ADC) and non-Gaussian parameters (goodness of fit R^2^>0.8) for the primary tumor overlaid on the DWI image with b = 0.

**Figure 4:**
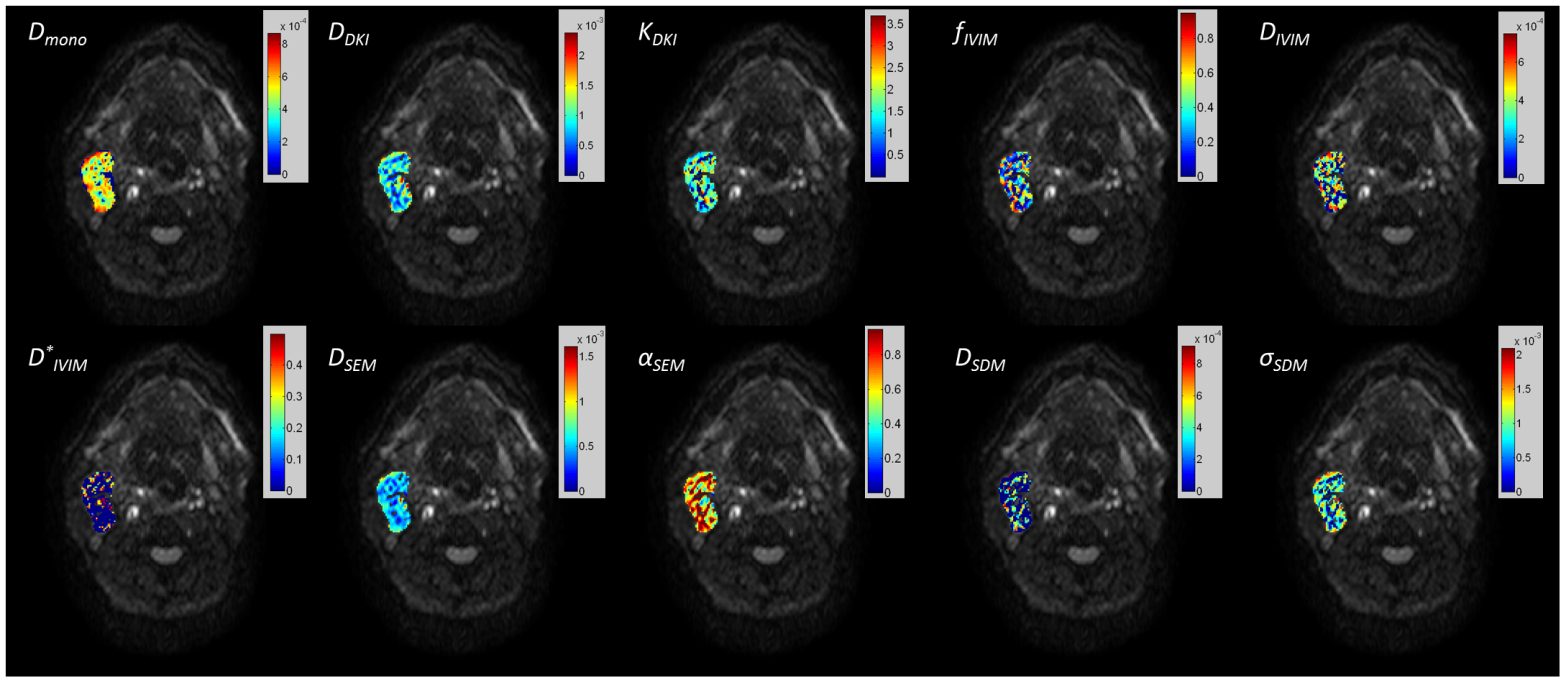
The maps of the mono-exponential apparent diffusion coefficient (ADC) and non-Gaussian parameters (goodness of fit R^2^>0.8) for the metastatic node overlaid on the DWI image with b = 0.

**Figure 5:**
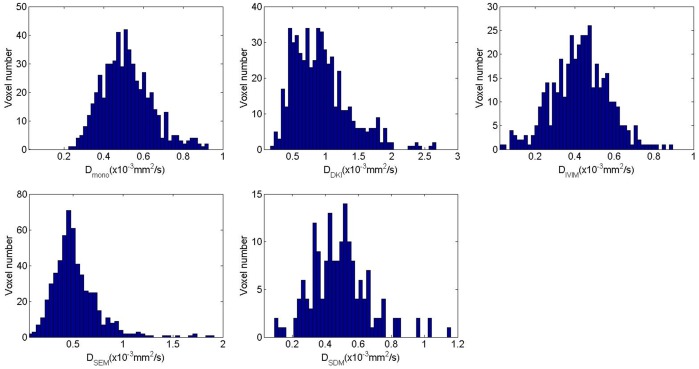
The histograms of the extracted voxel-wise diffusion coefficients based on the mono-exponential model (a), diffusion kurtosis model (b), intravoxel incoherent motion (c), stretched exponential model (d) and statistical diffusion model (e) from each model, i.e. *D_mono_, D_DKI_, D_IVIM_, D_SEM_* and *D_SDM_*, within the ROIs of the primary tumor in Fig. 3. Non-Gaussian diffusion coefficients show differences from the mono-exponential ADC in value and distribution pattern. Note that the fitted values around zero for the statistical model were excluded in (e) for better visualization.

**Figure 6:**
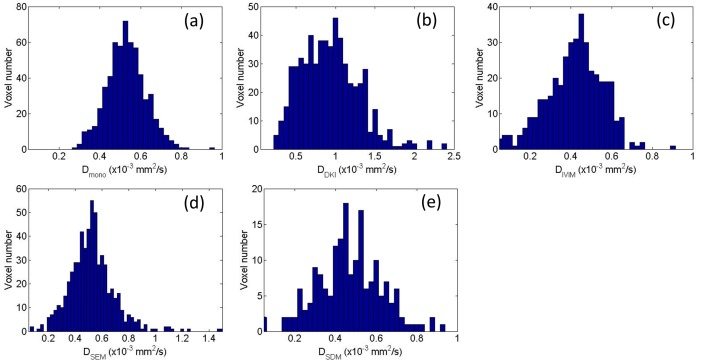
The histograms of the extracted voxel-wise diffusion coefficients based on the mono-exponential model (a), diffusion kurtosis model (b), intravoxel incoherent motion (c), stretched exponential model (d) and statistical diffusion model (e) from each model, i.e. *D_mono_, D_DKI_, D_IVIM_, D_SEM_* and *D_SDM_*, within the ROIs of the metastatic node in Fig. 4. Note that the fitted values around zero for the statistical model were excluded in (e) for better visualization.

Figures 3 and 4 depict the maps of the diffusion coefficients and other non-Gaussian model parameters (R^2^>0.8) extracted from different diffusion models for the primary tumor and the metastatic node, overlaid on the DWI image with b = 0 s/mm^2^, respectively. The histograms of the corresponding diffusion coefficients from each model, i.e. *D_mono_, D_DKI_, D_IVIM_, D_SEM_* and *D_SDM_*, within the ROIs of the primary tumor (Fig. 3) and the metastatic node (Fig. 4) are plotted in Fig. 5 and Fig. 6, respectively. For the maps of *D_SDM_*, more than half of voxels had the best fit values close to zero so these voxels were excluded in the histograms in Fig. 5e and Fig. 6e for better visualization (otherwise an extremely high bar would appear at zero). The diffusion coefficients extracted from the non-Gaussian models showed that there were substantial deviations from *D_mono_* (or ADC) both in magnitude and in distribution pattern. In particular, *D_DKI_* was larger than *D_mono_* while *D_IVIM_* was smaller than *D_mono_*. On the other hand, *D_SEM_* was relatively close to *D_mono_* but had a slightly wider value distribution at both histogram tails. In the primary tumor, *D_DKI_, D_SEM_*, *D_IVIM_,* and *D_SDM_* had a Spearman rank-order correlation factor (*r_Spearman_*) of 0.49, 0.64, 0.89, and 0.32 with *D_mono_* respectively. The corresponding Spearman rank-order correlation factors within the metastatic node were 0.39, 0.54, 0.91, and 0.52, respectively. All non-Gaussian diffusion coefficients showed positive correlations with the mono-exponential ADC but with different correlation strengths. *D_IVIM_* showed the strongest correlation while *D_DKI_* and *D_SDM_* showed relatively weak correlations with *D_mono_*. On the other hand, the diffusion kurtosis *K_DKI_* had a weak correlation with *D_DKI_* (*r_Spearman_*<0.4 in tumors and *r_Spearman_*<0.5 in nodes). Meanwhile, the stretching parameter *α_SEM_* also had a weak correlation with *D_SEM_* (*r_Spearman_*<0.3) in both primary tumors and metastatic nodes.

Table 1 summarizes the statistics of the mono-exponential ADC and non-Gaussian parameters for 13 primary tumors and 9 metastatic nodes. Mann-Whitney *U*-test showed that there were no significant differences between the extracted parameters from any DWI models for primary tumors and metastatic nodes.

**Table 1:** The statistics of the extracted mono-exponential ADC and non-Gaussian parameters for primary tumors and metastatic nodes.

	ADC_mono_ (×10^−3 ^mm^2^/s)	D_DKI_ (×10^−3 ^mm^2^/s)	K_DKI_	f_IVIM_	D_IVIM_ (×10^−3 ^mm^2^/s)	D^*^_IVIM_ (×10^−3 ^mm^2^/s)	D_SEM_ (×10^−3 ^mm^2^/s)	α_SEM_	D_SDM_ (×10^−3 ^mm^2^/s)	σ_SDM_ (×10^−3 ^mm^2^/s)
Primary Tumors	0.62±0.19	0.86±0.37	1.53±0.42	0.31±0.18	0.67±0.17	43.23±21.39	0.64±0.31	0.71±0.06	0.52±0.24	1.14±0.56
Metastatic Nodes	0.56±0.11	0.92±0.28	1.46±0.29	0.34±0.21	0.61±0.18	39.30±22.51	0.59±0.20	0.68±0.08	0.50±0.27	1.07±0.38

Data are reported as mean±SD. SD: standard deviation.

## Discussion

In this study, DWI images for head and neck were acquired in patients with NPC using an extended b-value range from 0 to 1500 s/mm^2^, and diffusion weighted signal decay was analyzed using mono-exponential and non-Gaussian diffusion models. The fitting results by using the non-Gaussian DWI models were compared with those by using the normal mono-exponential model.In all four tissues of interests in head and neck, namely primary tumor, metastatic node, spinal cord and muscle, diffusion attenuated signals showed substantial deviations from the mono-exponential decay within the applied b-factor range. Non-Gaussian diffusion models, with the exception of the SDM model, obtained significantly better goodness of fit for primary tumors, metastatic nodes, and muscles than the mono-exponential model, indicating non-Gaussian water diffusion behaviors at this extended b-value range up to 1500 s/mm^2^. The statistical diffusion model did not yield better DWI data fitting than the mono-exponential model. The predominant fitted values of *D_SDM_* close to zero may attribute to the ill-conditioning of the fitting procedure because of the relatively low signal-to-noise ratio (SNR) of DWI images. Mathematical analysis shows that least-square fitting to the SDM model imposes strict requirement on the extremely high signal SNR. A small variation of signal in the presence of noise could result in a large variability of the fitting result [38]. The reliability of SDM model fitting is expected to be improved by the enhancement of the DWI image SNR as well as the development of advanced fitting algorithms. For the spinal cord, the IVIM model showed significantly better fit than the mono-exponential model. It is understandable because IVIM is good for identifying pseudo-diffusion D^*^_IVIM_ reflecting the microcirculation or flow at low b values and true diffusion at high b values. Although the SEM model does not provide clear physiologic basis of the stretching parameter α_SEM_, our results indicated that SEM also obtained comparable goodness of fit as IVIM, and might be alternatively used for diffusion analysis in spinal cord as well. In this study, DKI achieved better DWI fitting than the normal mono-exponential model in primary tumors and metastatic nodes. However, DKI may not be good to describe the diffusivity for tissues in which flow or microcirculation perfusion effect is pronounced such as spinal cord. Another limitation of the DKI model is that the biological relevance of the diffusion kurtosis *K_DKI_* is still not fully understood.

Our results showed that non-Gaussian diffusion coefficient maps generated by voxel-by-voxel fitting were substantially different from ADC maps but with positive correlations. D_DKI_ was generally larger than D_mono_, possibly due to the second-order polynomial nature of the DKI model [39]. On the other hand, D_IVIM_ was smaller than D_mono_ because the contribution of flow or perfusion effect on diffusion was excluded. D_IVIM_ was relatively close to D_mono_ with high Spearman rank-order correlation coefficient. Considering that there were substantially different diffusion values as well as markedly different histogram distributions of non-Gaussian diffusion coefficients, these observations suggested that these measures might be used as potential complementary descriptors of tissue diffusivity for clinical applications. The low correlations of the stretching parameter α_SEM_ and diffusion kurtosis K_DKI_ with their corresponding non-Gaussian diffusion coefficients may also have potentials to reveal additional physiological properties of tissues beyond the diffusion coefficients. For voxel-by-voxel fitting using IVIM, D^*^_IVIM_ were systematically much larger than D_IVIM_ because of the larger displacement of water in the blood of capillary networks than the statistically averaged displacement of extracellular water in tissues during the application of diffusion gradients. However, the fitted D^*^_IVIM_ had much wider value distribution span than D_IVIM_. In principle, D^*^_IVIM_ and f_IVIM_ fitting is technically challenging and highly SNR demanded [36], [40], [41]. This may partially explain the heterogeneous maps of D^*^_IVIM_ and f_IVIM_.

This study has some limitations. This is a preliminary pilot study only involving limited numbers of NPC patients and lesions. Although we have shown that non-Gaussian DWI model better described the diffusion weighted signal decay in head and neck, the robustness of parameter estimation for some non-Gaussian DWI models, like IVIM and SDM, could be compromised by the least-square fitting and the relatively low SNRs of DWI images. The higher SNR is always preferable but usually leads to the longer scan time. More robust and reliable estimation algorithms are to be developed to improve the quantification accuracy and precision. In addition, the non-Gaussian DWI analysis usually involves the use of more diffusion-weighted images so could be time-consuming in DWI acquisition. In addition, the sampling strategy and protocol could also affect the parameter quantification performances for different non-Gaussian models [42], [43]. The b-factor choice in this study may not be optimal for all non-Gaussian models and should be further optimized to make a tradeoff between the minimization of scan time lengthening and parameter estimation reliability. For example, the b-values higher than 1000 s/mm^2^ are seldomly used for Gaussian diffusion analysis and ADC quantification, but are necessary in DKI [4]. The highest b-value applied in this study was 1500 s/mm^2^, the same as in [24] but still much smaller than those used in the brain [4], [9] to take in consideration the more pronounced susceptibility and lower SNR in the head and neck than in the brain. To mitigate the influence of low SNR on the quantification, a relatively high NSA of three was utilized for DWI scan at the cost of the lengthened acquisition time. After signal averaging, the SNRs for NPC primary tumors and metastatic nodes generally ranged between 10 and 20 at the highest b-factor of 1500 s/mm^2^. The accuracy of DKI analysis, particularly D_DKI_, might not be severely compromised at these SNRs. On the other hand, IVIM analysis more relies on the DWI signal at low b-factors to better quantify perfusion induced pseudo-diffusion D* and its fraction *f*. The number of low b-factors below 200 s/mm^2^ was limited (only three) in this study so the inclusion of more low b-factors should be helpful to improve IVIM quantification. Nevertheless, several recent studies also showed that IVIM analysis could even be feasible by using only three b-values, which substantially reduces the DWI scan time [44], [45]. Despite non-Gaussian diffusion models exhibited significantly better DWI data fitting in this study, the physiologic or pathologic basis for the non-Gaussian diffusion properties is still unclear and should be further elucidated.

In conclusion, diffusion characteristics of head and neck DWI signal decay at 3 Tesla in the patients with nasopharyngeal carcinoma were technically investigated by using various non-Gaussian diffusion models. Diffusion in NPC and other tissues in the head and neck exhibited apparent non-Gaussian behaviors. DWI signals could be significantly better fitted by non-Gaussian diffusion models compared to the normal mono-exponential model. Additional non-Gaussian parameters beyond ADC were able to be quantified and may potentially reveal additional tissue properties. Non-Gaussian diffusion analysis holds potentials to be used as a complementary tool to the normal DWI model but its diagnostic power and clinical merit should be evaluated in future studies.
